# Violence against Health-Care Workers in Governmental Health Facilities in Arar City, Saudi Arabia

**DOI:** 10.1155/2020/6380281

**Published:** 2020-03-20

**Authors:** Ruqayyah B. Al Anazi, Saeed M. AlQahtani, Amal E. Mohamad, Sabry M. Hammad, Hossam Khleif

**Affiliations:** ^1^Resident of Saudi Board of Family Medicine, Northern Borders General Health Affairs, Arar, Saudi Arabia; ^2^Family Medicine, King Abdul-Aziz Medical City, National Guard Heath Affairs, Riyadh, Saudi Arabia; ^3^Public Health and Community Medicine, Faculty of Medicine, Zagazig University, Zagazig, Egypt; ^4^Public Health & Community Medicine, Northern Borders General Health Affairs, Arar, Saudi Arabia; ^5^Public Health and Community Medicine, Faculty of Medicine, Mansoura University, Mansoura, Egypt; ^6^Family Medicine, Northern Borders General Health Affairs, Arar, Saudi Arabia

## Abstract

**Background:**

Violence against health-care workers (HCWs) showed increasing worldwide concern. No previous studies addressed violence against HCWs in the Northern region, Saudi Arabia.

**Objectives:**

To determine the prevalence of violence against HCWs in public hospitals and primary health-care centers in Arar city, KSA, and to identify its associated factors.

**Methods:**

A cross-sectional study was conducted on 352 HCWs in the Ministry of Health (MOH) facilities in Arar city from 1^st^ October to 31^st^ December 2018. Consented HCWs completed a structured self-administered questionnaire which was modified from the WHO questionnaire for violence.

**Results:**

Out of 352 health-care workers, 171 (48.6%) reported exposure to violence during work in the past year. The verbal violence was the most common form experienced (83%). Physicians were the main exposed group (59%). Being non-Saudi HCWs, older with longer duration of experience, working in hospitals, working in the emergency room, and working in evening or night shifts were significantly associated with more exposure to violence. The unmet demand for the patient and deficient staff number were the leading reasons for aggression. Only 16.4% of assaulted HCWs reported the violent acts to the higher health affairs authority with the most frequent reasons for nonreporting were their perception that it was useless and their fear of negative consequences.

**Conclusions:**

Violence against HCWs in Arar city, KSA, is a prevalent problem. Improving health security system and increasing staffing and their training on proper dealing with violence are highly recommended. Also, enforcing rules and regulations is an important demand to control and prevent violence against HCWs.

## 1. Introduction

Violence against health-care workers (HCWs) is becoming a major public concern noticeably. Repeatedly, different incidents of violence on health-care providers have been reported worldwide [1].

Workplace violence (WPV) is defined by the World Health Organization (WHO) as “incidents where staff are abused, threatened, or assaulted in circumstances related to their work, involving an explicit or implicit challenge to their safety, well-being, or health”. Also, definition of physical and verbal violence was adopted from WHO. Physical violence included beating, kicking, slapping, stabbing, pushing, biting, and pinching, while verbal violence or abuse was defined as being shouted at, sworn at, humiliated and threatened to harm, and use of indecent words [2].

Workplace violence (WPV) can affect the victim's health physically and mentally. It presents in all work environments, particularly health care. It compromises the good quality of care provided and affects the safety of health-care workers [3, 4].

On reviewing previous studies, the statistics of health-care violence are shocking. Nevertheless, they might not reflect the real picture of this issue. In Saudi Arabia, 65% of health-care professionals were victims of violence. The most frequent type of violence was verbal abuse. The aggressor can be the patient, patient's relatives, or coworker [5]. In another study in Riyadh, almost half of the nurses had been attacked, and the majority of participants perceived violence as verbal abuse. Shortage of staffing, misunderstandings, long waiting hours for service and lack of staff training, and policies for preventing crisis were reported as the main contributing factors [6]. In family medicine centers in Riyadh, it was reported that 48.0% of HCWs who were exposed to violence did nothing, 38.2% reported the incident, and 13.8% consulted a colleague [7].

In developed countries, researchers recommended strategies that prevent violence, and many prevention measures were implemented according to that, but in developing countries, this topic is rarely addressed [8].

This problem was not tackled or studied in Northern borders region, Saudi Arabia, so, this study was conducted to determine the prevalence, types, associated factors, consequences and suggested preventive measures of violence against HCWs in Arar city.

## 2. Subjects and Methods

A cross-sectional study was conducted in the MOH hospitals and Primary health-care centers of Arar city, KSA. There are three general hospitals and twelve primary health-care centers in Arar city. One of three hospitals and two out of twelve primary health-care centers were chosen randomly by the simple random sampling technique. All health-care workers in the selected facilities were included.

The sample size was calculated based on sample size formula [9]:

(1) $$\begin{array}{c} n={\left[\frac{z\alpha }{d}\right]}^{2}\times p\left[1-p\right], \end{array}$$

where *n* is the sample size, *zα* is the confidence interval taken as 1.96, *d* is taken as 0.05, and *p* is the probability in this study considered according to previous study conducted in Saudi public hospitals which reported that 67.4% of health-care professionals were victims of violence [10]. The minimum sample size calculated was 338. About 10% of the sample size was added to compensate for refusals to participate in the study. The sample size for the study was then 372. Twenty HCWs refused to participate in the study. Therefore, the total number of respondents was 352 with response rate of 94.6%.

All respondents were informed about the purposes of the study. The researcher used a self-administered questionnaire for data collection during the period from 1^st^ October to 31^st^ December 2018. The questionnaire was developed by authors by modification of the World Health Organization questionnaire on violence against HCWs [11]. The questionnaire had three parts. The first part collected participant demographic and occupational characteristics (i.e., age, gender, marital status, nationality, professional group, years of experience, and participation in shift work). The second part examined the respondent's awareness of procedures for violence reporting. It also included questions about the extent of worrying regarding WPV experiences, factors they think can lead to violence, and strategies that can help to prevent it. The third part examined respondents' experiences with violence. A pilot study was conducted on 30 health-care workers to test the clarity of the questions, validity, and reliability. No modifications were made in the questionnaire. They were included in data analysis.

### 2.1. Data Analysis

The researcher analyzed data using Statistical Package for the Social Sciences version 20 (SPSS, 20) software. Descriptive statistics were performed in the form of frequencies and percentages for all Categorical variables. Analytic statistics were conducted using the chi-square *χ*2 test for comparing categorical variables. Values of *P* ≤ 0.05 were considered significant.

### 2.2. Ethical Considerations

The researcher obtained ethical approval from the local research ethical committee of Northern Borders General Health Affairs, KSA (8/39), and administrative approval from hospital administration. The participants were oriented about the objectives of the study, and written informed consent was taken. They were assured that collected data will be treated with high confidentiality, and personal identifying information will not be published.

## 3. Results

A total of 372 HCWs were invited to participate in our study. Twenty of them refused to participate. Therefore, the total number of valid participants was 352 with response rate of 94.6%. The mean age of the studied health-care worker was 35.4 ± 8.5 years with a range of 21–62 years. Slightly less than two-thirds of responded HCWs were females (62.2%), and the majority was Saudi (61.4%). Out of the studied participants, 196 (55.7%) were working in hospital and the remaining were working in PHCs. The physicians and nurses constituted the majority of participants (80.4%), and other HCWs constituted only 19.6%. Approximately half of the participants (48.6%) had experienced violence at least once in the last year. The patients were more frequent perpetrators of violence among HCWs exposed to violence than the patient's relatives (71.9% and 48.5%, respectively).

Table 1 shows the prevalence of violence among participating HCWs in relation to their demographic and occupational characteristics. It was significantly associated with older age, being divorced or widow or being non-Saudi. The physicians were more exposed to violence than other HCWs, but the difference did not reach the level of significance. HCWs working in hospitals, in ER, and those having evening or night shift were more likely to be exposed to violent acts.

**Figure 1:**
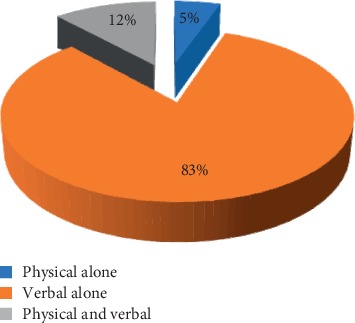
Distribution of HCWs exposed to violence (*n*=171) according to violence.

Regarding types of violence, 83% of total participants reported exposure to verbal violence, 5% reported exposure to physical violence, and 12% reported exposure to both verbal and physical violence (Figure 1).

The most important perceived factors contributing to violence were the unmet service demand, seeing expatriate as inferior and deficient staff number (Table 2). Regarding the measures suggested by studied HCWs to prevent exposure to violence, enhanced security system (64.8%) and increased staffing (62.8%) were the main recommendations (Table 3).

Table 4 illustrates consequences of exposure to violence among studied HCW. Nearly three-fourths of HCWs exposed to violence reported bothering mostly mild-to-moderately severe in the form of repeated, disturbing memories, thoughts or images of the violent attack, avoiding thinking or talking about the attack, being superalert, and feeling like everything you did was an effort.

Table 5 reveals that out of 171 health-care workers exposed to violence, only 28 (16.4%) reported exposure to the incident. The most common reasons for nonreporting were fear of negative consequences or feeling it is useless to report.

## 4. Discussion

Violence against HCWs is a well-recognized occupational hazard. This study was a cross-sectional study that aimed to explore the percentage and types of violence among HCWs working in public MOH facilities at Arar city, Saudi Arabia. In the current study, 48.6% of the participating HCWs reported exposure to violence at least once in the past year. This result is in line with that observed in previous studies conducted in Italy (45%) [12], India (47.02%) [13], Turkey (44.7%), [14] and Saudi Arabia (45.6%) [7]. However, a higher prevalence of violence against HCWs was reported in a study conducted in China (83.3%) [8], in Nepal (64.9%) [15], and in a study conducted in Riyadh city, KSA (67.4%) [10]. The difference in frequency of violence among HCWs may be due to difference in definition of violence, targeted professional groups of HCWS, and the methodology used.

As regards to the types of violence, the present study showed that the most frequent type of violence was verbal (83%), while physical violence represented only 5%. Earlier studies conducted in KSA reported similar finding as Algwaiz, and Alghanim reported (88.8% verbal and 1.6% physical) [10] and Al-turki et al. (94.3% verbal and 6.5%) [7]and in Bahrain by Rafeea et al. (78% verbal and 11% physical) [16]. A similar trend was also observed by other studies; however, the figures of verbal were much lower, and figures of physical were higher ([12, 15, 17, 18]). Verbal violence is more frequently reported than others because it is also an initial phase for subsequent physical violence. In the present study, none of the participants reported any acts of sexual harassment. This might be due to cultural sensitivity of this issue and fear of being stigmatized. This finding is similar to that of other studies [18, 19]. Some other studies reported exposure of the participants to sexual harassment: 1% in Turkey [14], 3% in Bahrain [16], 7.2% in Ethiopia [20], 8.6% in Palestine [21], and 11.3% in Nepal [15].

Emergency departments (EDs) have been recognized as an environment with high potential for workplace violence. Emergency personnel are more vulnerable to violence than other hospital personnel, perhaps due to their frontline nature of works and their 24-hour accessibility [21]. A study conducted in Ethiopia found that those working in EDs are four times more exposed to workplace violence than OPD workers. In ER settings, people come in a panic with serious injuries and life threatening health conditions that make them behave aggressively against HCWs. All these are aggravated by the anxiety and stress of HCWs due to high workload [20]. This aggressive behavior against health-care workers in ED has been observed by current study where ER HCWs were three times more likely than their counterparts in other departments to report violence (*P*=0.002, OR: 3.22, 95% CI: 1.45–7.16).

In an Ethiopian study, Yenealem et al. demonstrated that HCWs with less than 6 years of experience were three folds more likely to have experienced violence than their seniors with more than 16 years of experience. They explained this finding by the fact that young HCWs with short duration of experience are lacking the skills of managing violent acts that are acquired through experiences [20]. In a Turkish study, the risk of violence was 2.4 times higher among HCWs aged less than 30 years than older ones; however, the experience duration was not a significant risk factor for violence [14]. In Riyadh, KSA, two studies showed that less experienced and younger HCWs respondents were more likely to encounter violent attacks than their counterparts [5, 10]. Unexpectedly, the present study showed that older HCWs aged 50 years or more were 6 times more likely to experience violence than younger HCWs aged less than 30 years (*P* < 0.0001, OR = 6.24, 95% CI: 2.43–15.9). Similarly, those with longer duration of experience (10+ years) were more than two times likely to suffer violence than those working less than 5 years (*P*=0.002, OR = 2.41, 95% CI: 1.38–4.19). Alsaleem et al. noticed a similar finding in Abha city, Saudi Arabia, as they found older HCWs were associated with increased risk of violence by 3% more than younger ones (OR = 1.03; *P*=0.048) [18]. Also, El-Gilany et al. in Al-Hassa, KSA, found that HCWs with ten years' experience or more were more exposed to a higher risk of violence than those with less than five years of experience [19]. The same finding was also reported by other studies [8, 22].

Working in evening or night shift exacerbates the occurrence of violent acts among HCWs. Also, working in shifts disrupts the circadian rhythm of HCWs which increases their chance of development of physical illnesses and leads to fatigue. Inadequate security, few staff number, and low work performance due to fatigue during shift poses favourable conditions for violence. The current study indicated that HCWs who worked in the evening (*P* < 0.001, OR = 3, 95% CI: 1.59–5.64) or night shifts (*P* < 0.0001; OR = 9.3; 95% CI: 3.5–24.68) were significantly more at risk of violence than their colleagues who worked in the morning. This finding is consistent with that reported in other studies [8, 18, 20].

Different studies revealed different reasons contributing to workplace violence (WPV) against HCWs. Kumar et al., found that long waiting periods (73.5%) and delay in medical care provision (45.7%) were the most common causes of the violence [13]. Algwaiz and Alghanim in their study in Saudi public hospitals indicated that increased waiting time (51.6%), shortage of staff (39.1%), and unmet patient's demand (38.0%) were the most frequent triggers of violence [10]. In Abha city, AlSaleem et al. found long waiting time (56%), staff shortage (52%), overcrowding (48%), workload (42%), and lack of security (41%) were the main reasons for the high prevalence of workplace violence [18]. El-Gilany et al., in their study in Al-Hassa, concluded that the main factors contributing to WPV were unmet service demand (72.2%), lack of penalty for the perpetrators (67.2%), overcrowding (65.9%), impatience of patients and their relatives (58.9%), and reaction to injury or illness (57%) [19]. Similarly, our study pointed that the most frequent causes contributing to WPV as perceived by studied HCWs were unmet service demand (69.3%), seeing expatriate as inferior (61.1%), and deficient staff number (52.2%).

In the present study, out of 171 exposed to violence, 83.6% did not report the violent incident. Nonreporting was also observed in other studies [5, 17]. A variety of reasons for nonreporting of violent acts was demonstrated by many studies [5, 7, 21, 23]. In our study, the majority (92%) were either worried about negative consequences such as revenge of perpetrators or believed that it was useless to report. It was striking that 35% of the studied sample was unaware of reporting procedures. Al-Turki et al., in their study in family medicine centers in Riyadh city, found that underreporting of violence by HCWs was caused by their belief that reporting was useless and some feared losing their jobs [7]. In another two Saudi studies, the participants considered reporting as useless or unimportant [5, 23]. In an Iranian study, the victimized HCWs felt that reporting was of little value since nothing would be done [24].

With regard to consequences of exposure to violence, the present study showed that nearly three fourths of the participants reported bothering mostly mild to moderately severe in the form of repeated, disturbing memories, thoughts or images of the violent attack, avoiding thinking or talking about the attack, being superalert, and feeling like everything you did was an effort. A similar finding was reported by El-Gilany et al., who found violence against HCWs had many consequences and the most frequent were becoming bothered, suspicious, anger, dissatisfied with work, irritable, anxious, and superalert [19].

The mostly suggested measures by the studied respondents for prevention and control of violence against HCWs were enhancing the security system in the health facilities (64.8%) and increasing staff numbers who provide health care (62.8%). Kumar et al. reported a similar finding [13]. In Al-Hassa's study, in Saudi Arabia, the presence of security personnel, communications with police, and increased penalties for offenders were the most frequently suggested factors to minimize the violent incidents against HCWs [19].

Although many studies have been conducted on violence against health-care workers in different settings worldwide with a different culture, all agreed there is an urgent need to address the increased prevalence of aggression against HCW and to ensure safe working environment preserving both patient and health-care workers' rights.

## 5. Conclusion

Violence against HCW is a common problem facing HCWs. Most of the violent attacks were verbal with many negative consequences. These findings highlight the need for a comprehensive approach for prevention and control of WPV in health facilities. Saudi health-care policy planners should consider policies, measures, and regulations that protect health-care workers. Improving security system in health facilities is a necessity for minimizing the violence incidents. Also, increasing staffing is important as it will lead to rapid delivery of health services, and thus violence due to long waiting times will be prevented. Moreover, training of HCWs on how to improve their coping skills when they are exposed to violence is urgently needed. Furthermore, increasing the awareness of the public about the important role of HCWs profession in the continuity of providing health-care services is required.

### 5.1. Limitations

Our study has some limitations. Firstly, it relies on self-reported data which are liable to bias. Secondly, the participants were inquired about exposure to violence in the previous year which might be subjected to recall bias. Lastly, the study included only public MOH facilities in Arar city ignoring effects of private facilities. Therefore, the results cannot be generalized to HCWs in Saudi Arabia.

## Figures and Tables

**Table 1:** Violence against studied health-care workers in relation to their demographic and occupational characteristics.

Characteristic	Total N (*n* = 352)	Violence (*n* = 171)	*P* value	Odds ratio	95% CI
		*N* (%)			
*Gender*					
Female	219	100 (45.7)	0.187	1^r^	—
Male	133	71 (53.4)		1.36	(0.88–2.099)

*Age*					
<30	114	37 (32.5)		1^r^	—
30–39	134	59 (44.0)	0.06	1.64	(0.97–2.75)
40–49	76	54 (71.1)	<.0001	5.11	(2.7–9.6)
50+	28	21 (75.0)	<.0001	6.24	(2.43–15.9)

*Marital status*					
Single	54	28 (51.9)		1^r^	—
Married	267	118 (44.2)	0.3	0.74	(0.41–1.32)
Divorced, separated, and widowed	31	25 (80.6)	0.008	3.87	(1.37–10.93)

*Nationality*					
Saudi	216	84 (39.4)	<.0001	1^r^	—
Non-Saudi	136	87 (64.0)		2.79	(1.79–4.35)

*Health facility*					
PHC	156	52 (33.3)	<.0001	1^r^	—
Hospital	196	119 (60.7)		3.09	(1.99–4.79)

*Department*					
Outpatient	241	114 (47.3)		1^r^	—
Inpatient	76	31 (40.8)	0.32	0.77	(0.455–1.29)
ER	36	26 (74.3)	0.002	3.22	(1.45–7.16)

*Professional category*					
Physician	122	72 (59.0)	0.06	1.77	(0.97–3.20)
Nurse	161	68 (42.2)	0.71	0.89	(0.51–1.58)
Others	69	31 (44.9)		1^r^	—

*Years of experience*					
<5	86	33 (38.4)		1^r^	—
5-<10	131	57 (43.5)	0.45	1.24	(0.71–2.16)
10+	135	81 (60.0)	0.002	2.41	(1.38–4.19)

*Shift type*					
No shift	265	106 (40.0)		1^r^	—
Evening shift	51	34 (66.7)	0.001	3.0	(1.59–5.64)
Night shift	36	31 (86.1)	<.0001	9.3	(3.50–24.68)

CI: confidence interval; *r*: reference category.

**Table 2:** Perceived factors leading to violence among studied HCW (*n* = 352).

Factors	*N*	%
‐Unmet service demand	244	69.3
‐See expatriate as inferior	215	61.1
‐Deficient staff	185	52.5
‐Long waiting time	156	44.3
-Overcrowding	148	42.0
-Inappropriate staff behavior	145	41.2
‐Impatience (patient in a hurry)	116	33.0
-Hot climate	85	24.1
‐Poor administration	40	11.3
‐Relatives of directors/managers	35	9.9

**Table 3:** Measures suggested by studied HCW (*n* = 352) to prevent exposure to violence.

Measures	*N*	%
-Enhance security system	228	64.8
-Increase staffing	221	62.8
-Training on violence prevention and control	123	34.9
-Better labeling	94	26.7
-Change policies to allow the victim to leave violence scene	81	23.0
-Liaison with police or emara (local authority)	76	21.6
-Changing the work environment and flow	67	19.0

**Table 4:** Consequences of exposure to violence among studied HCW (*n* = 171).

Bothering	Not at all	Mild to moderate	Extremely
	*N* (%)	*N* (%)	*N* (%)
Repeated, disturbing memories, thoughts, or images of the attack	45 (26.3)	108 (63.2)	18 (10.5)
Avoiding thinking or talking about the attack	40 (23.4)	105 (61.4)	26 (15.20)
Being superalert	38 (22.2)	97 (56.7)	36 (21.1)
Feeling like everything you do is an effort	42 (24.6)	101 (59.1)	28 (16.3)
Affect the way you deal with pt./colleagues in the future	62 (36.3)	79 (46.2)	30 (17.5)

**Table 5:** Reasons for nonreporting violence incident among HCW exposed to violence (*n* = 143).

Reasons	*N*	%
-Afraid of negative consequences	60	41.96
-Useless	60	41.96
-Did not know who to report to	51	35.7
-Felt guilty	45	31.5
-It was not important	29	20.3
-Felt ashamed	26	18.2

*Note.* Total is not cumulative.

## Data Availability

The data that support the findings of this study are available from the corresponding author, (A.E. Mohamed), upon reasonable request.
